# Whole Exome Sequencing Identified a Novel Biallelic *SMARCAL1* Mutation in the Extremely Rare Disease SIOD

**DOI:** 10.3389/fgene.2019.00565

**Published:** 2019-06-18

**Authors:** Jing Jin, Keke Wu, Zhenwei Liu, Xiaomin Chen, Shan Jiang, Zhen Wang, Weixing Li

**Affiliations:** ^1^School of Laboratory Medicine and Life Sciences, Wenzhou Medical University, Wenzhou, China; ^2^Wenzhou Center for Disease Control and Prevention, Wenzhou, China; ^3^Institute of Genomic Medicine, Wenzhou Medical University, Wenzhou, China; ^4^Center of Scientific Research, The Second Affiliated Hospital and Yuying Children’s Hospital of Wenzhou Medical University, Wenzhou, China; ^5^Research Center of Blood Transfusion Medicine, Education Ministry Key Laboratory of Laboratory Medicine, Zhejiang Provincial People’s Hospital, People’s Hospital of Hangzhou Medical College, Hangzhou, China; ^6^Zhejiang Center for Clinical Laboratory, Zhejiang Provincial People’s Hospital, People’s Hospital of Hangzhou Medical College, Hangzhou, China

**Keywords:** Schimke immuno-osseous dysplasia (SIOD), *SMARCAL1*, whole exome sequencing, mild phenotype, bioinformatics

## Abstract

Schimke immuno-osseous dysplasia (SIOD) is an extremely rare autosomal recessive pleiotropic disease. Although biallelic mutations in *SMARCAL1* gene have been reported to be the genetic etiology of SIOD, its molecular diagnosis has been challenging in a relatively proportion of cases due to the extreme rarity. Here, we made a definitive SIOD diagnosis of a 5-year-old girl with an extremely mild phenotype by applying whole exome sequencing (WES). As a result, a novel maternal mutation (c.2141+5G > A) confirmed to create a novel splice donor site combined with a known paternal mutation (c.1933C > T; p.Arg645Cys) were detected. In addition, previous reported SIOD cases showed excessive enrichment for mutations in the helicase ATP-binding and C-terminal domains of SMARCAL1. Similarly, the novel mutation we identified caused a mutant protein truncated in the SMARCAL1 C-terminus. Interestingly, based on the phenotypic profile, compared to reported cases, the patient in our study exhibited milder symptoms with renal dysfunctions limited to asymptomatic proteinuria, but no neurological signs or recurrent infections. Moreover, we identified 73 *SMARCAL1*-interacting genes, which formed a significant interconnected interaction network with roles in disease-related pathways such as double-strand break repair via homologous recombination, DNA repair, and replication fork processing. Notably, the top 15 *SMARCAL1*-interacting genes all showed a similar renal temporal expression pattern. Altogether, to our knowledge, the case in this study is the first case diagnosed originally based on a genetic test via WES rather than a characteristic phenotype. The identification of the novel allelic mutation (c.2141+5G > A) extends the phenotypic spectrum of *SMARCAL1* mutations and the following bioinformatics analysis presents additional genetic evidence to illustrate the role of *SMARCAL1* in SIOD.

## Introduction

Schimke immuno-osseous dysplasia (SIOD; OMIM #242900) is a rare autosomal recessive disorder with an estimated incidence of 1 in 3 million live births in the United States (Santangelo et al., 2014), which was first described by Schimke et al. in 1971 (Schimke et al., 1971). SIOD is a multisystem disease, the main clinical features of which include growth failure, spondyloepiphyseal dysplasia, progressive nephropathy, and poor cellular immunity (Saraiva et al., 1999; Boerkoel et al., 2000). Hypothyroidism, bone marrow failure, and episodic cerebral ischemia have also been reported in some severely affected patients (Boerkoel et al., 2000). Phenotypic abnormalities are another feature of SIOD patients and usually consist of a broad nose, lumbar lordosis, and a protruding abdomen. Many patients also have microdontia, hypodontia, or malformed deciduous and permanent molars along with hyperpigmented macules located on the trunk (Saraiva et al., 1999; Morimoto et al., 2012).

The SIOD disease is frequently reported to be caused by biallelic mutations in the gene *SMARCAL1* (SWI/SNF-related, matrix associated, actin-dependent regulator of chromatin, subfamily A-like 1), a member of the SNF2 family of proteins that regulates gene transcription, DNA replication, repair and recombination in the context of chromatin (Bansbach et al., 2010). *SMARCAL1* was found to play an important role in replication fork restarts, cell cycle progression, and DNA damage responses (Ciccia et al., 2009). Various mutations have been found to be distributed throughout the whole gene (Lipska-Zietkiewicz et al., 2017). Notably, nonsense or frameshift mutations of *SMARCAL1* can lead to a severe phenotype (Boerkoel et al., 2002), and biallelic missense mutations within the SNF2 domain in other family members were also found to affect protein subcellular localization, enzymatic activity, abundances and chromatin binding, etc. (Elizondo et al., 2009).

Schimke immuno-osseous dysplasia is a phenotypic heterogeneous disease with a marked variation in severity of clinical manifestations. Based on the severity, SIOD has been classified into two different subtypes: severe and mild (Ehrich et al., 1995; Saraiva et al., 1999). Patients with the severe subtype mostly suffer from recurrent infections and develop renal failure or cerebrovascular disease in the first 2 to 5 years of life (Yue et al., 2010). The diagnosis of SIOD mainly relies on characteristic clinical and radiographic features followed by molecular testing (mainly single-gene testing and a multigene panel) if the clinical evidence is insufficient (Lipska-Zietkiewicz et al., 2017), while whole exome sequencing (WES) has seldom been applied to detect *SMARCAL1* mutations (Morimoto et al., 1993).

In this study, we are trying to make a diagnosis of a 5-year-old girl with mild SIOD phenotypes, and in which it was difficult for the pediatrician to make a definitive diagnosis based on phenotype features only. By applying WES technology, one novel mutation (c.2141+5G > A) adjacent to the 5′ donor splice site predicted to disrupt splicing function and one well-known mutation (c.1933C > T; p.Arg645Cys) were identified in the *SMARCAL1* gene. Based on the results of genetic testing, we clarified an SIOD diagnosis of the 5-year-old girl, and the diagnosis was supported by the following series of abnormal clinical symptoms. Compared to previously reported patients who were diagnosed according to characteristic phenotypes with missense mutations at the same position of the Arg645 amino acid, our patient shows the mildest phenotype with no recurrent infections or neurological signs and extremely mild renal dysfunction. Our comprehensive bioinformatics analysis presented in this study further demonstrated the functional role of *SMARCAL1* in SIOD.

## Materials and Methods

### Patient Recruitment

This study was conducted in accordance with the guidelines of the Declaration of Helsinki. It was also approved by the Ethics Committee of the Second Affiliated Hospital and Yuying Children’s Hospital of Wenzhou Medical University. Written informed consent was obtained from both parents of the patient. SIOD was diagnosed according to the results of a genetic test ordered by an experienced pediatrician. Routine clinical and laboratory examinations, including hematological and immunological tests and skeletal X-ray, were performed on the patient to confirm the diagnosis.

### Whole Exome Sequencing and Variant Calling

Genomic DNA was extracted from the peripheral blood of the patient according to standard procedures using the QIAGEN DNeasy Blood & Tissue Kit (Qiagen, Valencia, CA, United States) and then sheared to 150 bp fragments in length. Whole-exome capture using the Agilent SureSelect Human All Exon v5 Kit (Agilent Technologies, Santa Clara, CA, United States) and high-throughput sequencing by utilizing an Illumina HiSeq 4000 sequencer (Illumina, San Diego, CA, United States) were conducted.

Computationally efficient read preprocessing and quality control for high-throughput sequencing data sets were taken with adaption of a canonical pipeline (Wang et al., 2013). The Trim Galore program was used to remove low-quality reads and adapters. The filtered reads (Phred-scaled quality score ≥ 30 and read length ≥ 80 bp) were aligned to the human reference genome (GRCH37/hg19) with the Burrows-Wheeler Alignment Tool (BWA) pipeline. Picard was then utilized to realign the reads from the BAM files and label the duplicated reads. In addition, local realignment and map quality score recalibration were performed. All variants, including single-nucleotide variants (SNVs) and InDels, were called according to three incorporated GATK tools: RealignerTargetCreator, IndelRealigner, and BaseRecalibrator. *De novo* and biallelic mutations were identified using mirTrios (Li et al., 2015).

### Variant Annotation and Prioritization

The ANNOVAR software tool and in-house codes were used to annotate all of the mutations (Wang et al., 2010). Based on phenotype-related databases including HGMD, any variant was assessed as to whether it was associated with a previously recorded phenotype. To detect rare mutations, variants with a minor allele frequency (MAF) > 0.1% in various publicly available variant databases, including ExAC, 1000 Genome, dbSNP and ESP were filtered out. Subsequently, the effects of the detected variants were predicted by four software tools: STFT (Kumar et al., 2009), Polyphen (Adzhubei et al., 2010), LRT (Chun and Fay, 2009), and CADD (Kircher et al., 2014). After Sanger sequencing confirmation, the remaining variants were thought to be high-confidence causative variants.

### RNA Exaction and Exon Junction Detection of cDNA

Total cellular RNAs from the Patient and control were isolated from blood samples using the TRIzol reagent (Invitrogen). And 1 μg of total RNA was used for reverse transcription with Reverse Transcription System (Promega) using oligo-dT primers. *SMARCAL1*-specific PCR primers were designed to amplify the region spanning the exon 13. Primer sets for hypothetical exon junctions were as follows: 5′-GCTGCAGCCAAGGAAATGAC-3′ in exon12-F and 5′-GCGTCCAGGACCACCTTATG-3′ in exon14-R. The final PCR products were visualized with electrophoresis, and submitted for Sanger sequencing as well.

### Construction of Physical Interaction Network and Biological Process Enrichment Analysis by DAVID Bioinformatics Resources

The direct protein–protein interaction (PPI) data set used in this study was downloaded from the STRING database. We extracted genes interacting with *SMARCAL1* in the STRING database with interaction scores greater than 400 to construct an internal interaction network. To evaluate the significance of the observed network, we performed random simulations of 100,000 iterations for genes and their connections selected from the STRING database. To further research the function of the genes in the PPI network, biological processes (BPs) analysis of gene functional analysis was conducted on the David website^1^.

## Results

### Clinical Presentation

The index patient, a 5-year-old girl from Han Chinese population, was the first child born to healthy, non-consanguineous, Chinese parents. The patient was referred to the Second Affiliated Hospital and Yuying Children’s Hospital of Wenzhou Medical University due to nasosinusitis and adenoid hypertrophy. Physical examination on admission demonstrated a disproportionate, short stature with a collapsed nose, spherical nose tip, short neck and a protruding abdomen. Multiple pigmented nevi were present on the abdomen and bilateral ankle. According to these clinical features, it was difficult to make a diagnosis to explain these abnormal symptoms. Therefore, after the consent of the patient’s parents, WES was used to perform genetic testing to detect possible genetic lesions.

**FIGURE 1:**
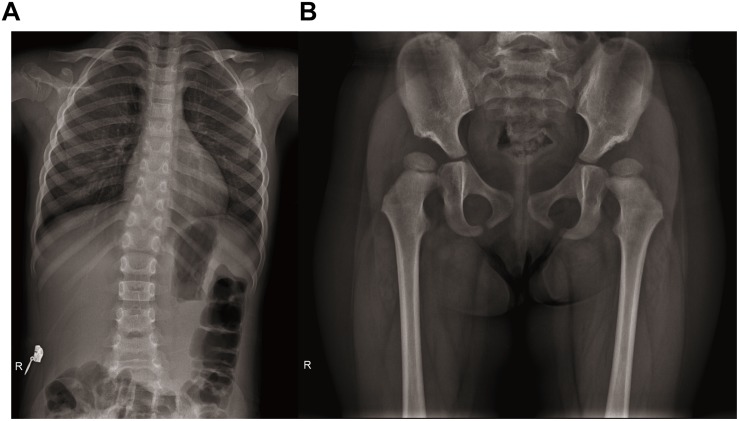
Radiographs of spine and hip joints. **(A)** The spine radiograph showing the patient has scoliosis. **(B)** Bilateral femoral head moved upward. Bilateral acetabular articular surface was flat and shallow. Bilateral Shen Tong’s line was discontinuous. Poor alignment of left hip joint.

After the genetic testing, the child patient was also hospitalized to complete a full physical examination for more phenotype features. Skeletal X-ray showed scoliosis (Figure 1A), hypoplasia pelivis (Figure 1B), and spondyloepiphyseal dysplasia with ovoid and flat vertebrate (Table 1). Although a quantitative test of 24 h urinary protein was 120.4 mg/L, which was in the normal range (<150 mg/24 h), renal dysfunction was evidence with an increased content of urinary microproteins, including microalbuminuria 5.82 mg/dl (<1.9 mg/dl), transferrin 0.45 mg/dl (<0.2 mg/dl), and IgG 1.13 mg/dl (<0.8 mg/dl) (Pomerance, 1997) (Table 1). Hematological and immunological studies showed a decreased ratio of lymphocytes 0.167% (0.23–0.53%), along with an increased ratio of neutrophils 0.724% (0.35–0.65%), and monocytes 0.10 (0.03–0.08%). The T lymph ratio was 43% (55–84%), indicating T cell immunodeficiency, including low levels of T helper and T suppressor lymphocytes (Table 1). Furthermore, dental examination found enamel dysplasia. The thyroid stimulating hormone content in her serum was 6.0067 mIU/L (0.8–5.0 mIU/L), confirming the diagnosis of hypothyroidism. Nevertheless, electrocardiogram, abdominal ultrasound, and skull MRI plain scans showed no abnormalities.

**TABLE 1:** Clinical features of the patient.

**Clinical features**		**Current situation**	**Characterization and indicators**
Development	Intrauterine growth retardation	+	Born at 36 weeks, weight of 1.25 kg, height of 40 cm (<3rd percentile)
	Microsomia	+	103.5 cm at 6-years old
Skeletal feature	Short neck	+	
	Short torso	+	
	Scoliosis	+	
	Oval flat vertebra	+	
	Pelvic hypoplasia	+	Bilateral femoral head moved upward, bilateral acetabular articular surface was flat and shallow, bilateral Shen Tong’s line was discontinuous. Poor alignment of left hip joint
	Abnormal femoral head	+	
Kidney dysfunction	Urine protein	+	MA: 5.82 mg/dl ( < 1.9 mg/dl)
	Kidney disease	−	
	Focal segmental glomerulosclerosis	−	
Abnormity of blood	T cell deficiency	+	CD3: 17%(62.0–70.0%), CD4: 11% (30.0–40.0%), CD8: 4% (20–27%)
	Lymphopenia	+	LY#: 1.00 × 109/L (0.92–5.3), LY%: 0.167 (0.23–0.53)
	Neutrophil reduction	−	NE#: 4.33 × 10^∧^9/ (1.4–6.5), NE%: 0.724 (0.35–0.65)
	Thrombocytopenia	−	PLT: 350 × 10^∧^9/L
	Anemia	−	HGB: 135 g/L (110–190)
Physical characteristics	Broad nose	+	
	Wide and collapsed bridge of the nose	+	
	Prominent belly	+	
	Multiple pigmented nevi	+	
	Abnormity of hair	+	
	Small or missing teeth	+	
	Corneal opacity	+	A small amount of granular gray-white reflection in the double corneal stroma is located in the posterior stromal layer of the bismuth.
Development	Hypoevolutism	−	
	Academic delay	−	
Vasculature	Headache	−	
	Transient cerebral ischemia stroke		No obvious abnormality of Skull MRI scan
Other inspections	Hypothyroidism	+	TSH:6.0067 mIU/L (0.8–5.0)
	Catarrhal dysentery	−	Fat globule:(+)
	Non-Hodgkin lymphoma	−	

*MA, microalbuminuria; LY, lymphocyte; NE, neutrophile granulocyte; PLT, blood platelet; HGB, hemoglobin; TSH, thyroid stimulating hormone. +, positive for the finding; −, negative for the finding.*

### Sequencing Data Analysis and Identification of Mutations

**FIGURE 2:**
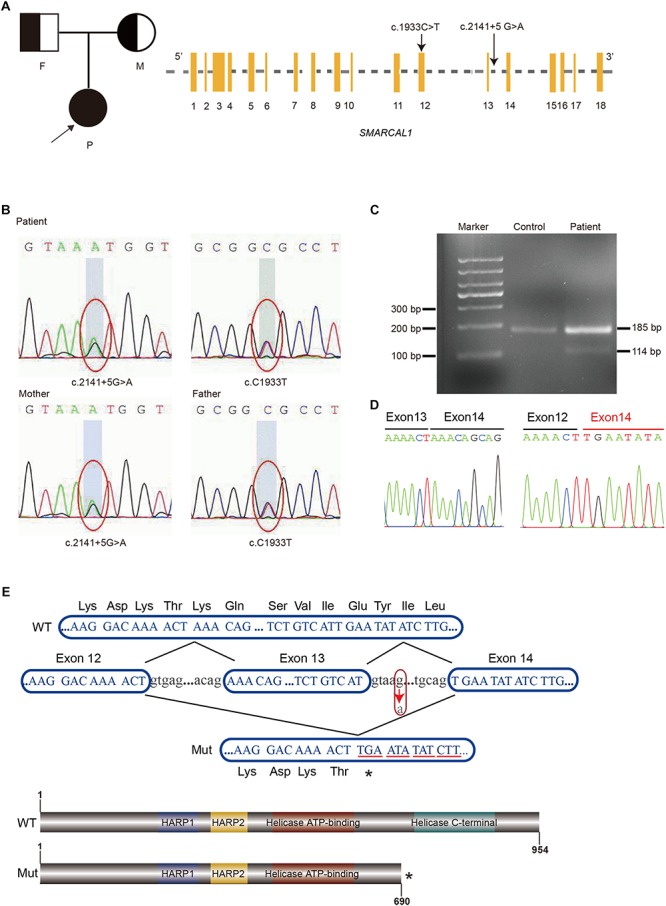
Identification of compound heterozygous mutations in *SMARCAL1.* **(A)** Pedigree of the Family and Schematic of *SMARCAL1*. Yellow boxes represent the coding exons; black characters indicate the mutation identified in this study. **(B)** Sanger sequencing confirmed the heterozygous mutations in *SMARCAL1.* The father was a carrier of the c.C1933T mutation and the mother was a carrier of the c.2141+5G > A mutation. **(C)** Reverse transcriptase PCR analysis on cDNAs from patient and control. An abnormal PCR product (114 bp) was detected in patient. **(D)** Sanger sequencing for the fragment amplified from the cDNAs of both patient and control. **(E)** Nucleotide sequences from exon 12 to exon 14 of *SMARCAL1* and protein schematic of smarcal1. WT, wild type; Mut, mutant. The arrow indicates the c.2141+5G > A variant. The asterisk means a premature stop codon.

Although the preliminary examinations showed disproportionate short stature, special facial dysmorphism, hypothyroidism and multiple pigmented nevi, based only on these observations, it was hard to make a diagnosis. Considering the possibility of genetic defects, WES of the patient was performed. After rigorous screening of mutations and Sanger sequencing validation, a known exon mutation (c.1933C > T, p.Arg645Cys) and a new intron mutation (c.2141+5G > A) adjacent to a splicing region (Figure 2A) were identified in the *SMRACAL1* gene. Haplotype analysis and sequencing showed that the c.1933C > T mutation was inherited from father and that the c.2141+5G > A mutation was from mother (Figure 2B).

The previously reported mutation Arg645Cys was predicted to be pathogenic by four commonly used bioinformatic algorithms, including SIFT (damaging), polyphen-2 (probably damaging), LRT (deleterious), and CADD (damaging). In addition, the amino acid residue was predicted to be conserved. Meanwhile, this variant has shown a low frequency of 8.25 × 10^–6^ in the ExAC database and was absent in the UK10K, 1000 Genomes and ESP6500. In accordance with the recommended standards of the American College of Medical Genetics (ACMG) (Richards et al., 2015), the variant c.1933C > T is categorized as a “pathogenic variant” because of its classification as PS1, and PS3 (Supplementary Table S2). According to the annotation, the c.2141+5G > A mutation has not been previously observed in any public database.

### The Novel c.2141+5G > A Allele Affects the Splicing of *SMARCAL1*

The c.2141+5G > A site was adjacent to the 5′ donor splice site in intron 13 (Figure 2A and Supplementary Figure S1) and was confirmed in the patient and her mother. Though it was a variant of an intron region, we hypothesized that it was the second allele as a compound mutation that caused SIOD, an autosomal recessive multisystem disorder. The mutation was not previously observed in various publicly available databases, including ExAC, UK10K, dbSNP147, 1000 Genomes, or the ESP6500 database. We used four splicing prediction tools dbscSNV (Jian et al., 2014), Human Splicing Finder (Desmet et al., 2009), MaxEntScan (Yeo and Burge, 2004), and SPANR (Elliott et al., 1976) to predict splicing changes, which resulted in the creation of a novel splice donor site (Supplementary Table S3). Splicing mutations can lead to a mixture of aberrantly and correctly spliced transcripts by partial skipping of exons or inclusion of intronic sequences, or they can change the ratio of programmed alternatively spliced isoforms (Nissim-Rafinia and Kerem, 2005). The position of the mutation immediately adjacent to the 5′ donor splice site suggested it might disrupt 5′-splice site function, result in the skipping of contiguous exon from the mature mRNA and lead to a truncated protein (Montalban et al., 2018).

The exact impact of the c.2141+5 G > A variant on splicing was confirmed by RT-PCR with total RNA extracted from the blood samples from the patient and control, using forward and reverse primers located in exons 12 and 14 respectively. Two different amplification products were detected from the *SMRACAL1* cDNA of the patient: the upper band derived from the full-length transcript which was corresponded to the wild-type allele (Figure 2C), whereas the lower band derived from an aberrant transcript lacking the entire exon 13 (c.2070_2141de71) (Figure 2C). This exon skipping lead to premature stop codon at position 260 that results in the loss of 247 amino acids from the SMARCAL1 C-terminus, resulting in the loss of Helicase C-terminal functional region (Figures 2D,E). According to the guide of ACMG (Richards et al., 2015), the novel variant c.2141+5G > A would be classified as a “pathogenic variant” supported by the classification as PVS1, PM2, and PM3.

### Phenotypic Profile and Mutation Summary

To gain a global understanding of the various disease phenotypes and mutations, we collected all of the previously reported cases based on abundant literature. SIOD has a marked variation in severity, ranging from in utero onset with growth retardation and death within the first 5 years of life to onset of symptoms in late childhood. Based on existing phenotypic data, most of the patients had symptoms of spondyloepiphyseal dysplasia (97.5%), T lymphocyte decrease (85.96%) and maculas (62.67%) (Supplementary Table S1), while some of the patients diagnosed severe SIOD had blood pancytopenia, recurrent infections and central nervous symptoms (Supplementary Table S1).

**FIGURE 3:**
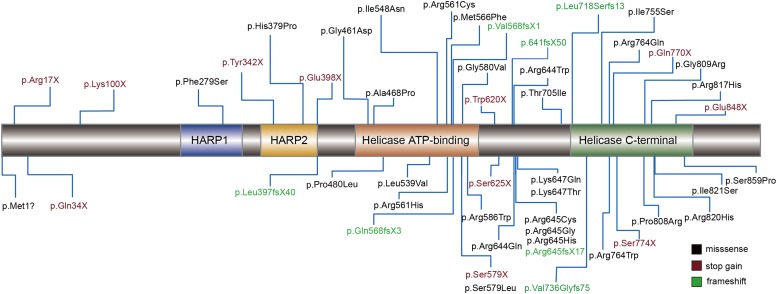
Schematic diagram of mutations assiociated with SIOD in the encoded proteins of *SMARCAL1* gene. Rectangles with different colors indicate specific protein domains. Black font represents missense mutation, vermeil font shows stop-gain mutations, and green font indicates frameshift mutations.

Schimke immuno-osseous dysplasia is a genetically heterogeneous disease. The pathogenicity of the mutation was usually considered to cause a change in protein function. Therefore, we mapped 49 of the reported amino acids alternative mutations to the schematic representations of SMARCAL1 (Figure 3). Helicase ATP-binding and helicase C-terminal domains showed excessive enrichment of mutations. The Arg645 site tends to be a recurrent susceptible locus of SMARCAL1 with diverse amino acid changes, and the other biallelic site (c.2141+5G) is close to exon 13, which encodes a part of the helicase C-terminal subdomain. Besides, we also mapped all the pathogenic mutations in intron regions to the nucleotide structure map of *SMARCAL1* (Supplementary Figure S1), among which a reported homozygousmutation c.2244+5 G > A in intron 14 detected in a SIOD patient with mild developmental delay and mild ID was near the novel c.2141+5G > A mutation in this study.

Notably, the variants at residue Arg645 was previously reported as a compound mutation in four SIOD patients (Table 2). All the patients were diagnosed with mild SIOD based on mild multisystem clinical symptoms, while the definite diagnosis of our patient was only made after genetic testing. Compared to the four patients that carried missense mutations at the same locus, our patient had milder symptoms without any neurological signs and recurrent infections. Remarkably, the renal dysfunctions of our patient were limited to asymptomatic proteinuria while the kidney histopathological examination of the other four patients showed varying degrees of abnormalities. In addition to the mildest symptoms, the diagnostic age of our patient was the youngest.

**TABLE 2:** Genotype–phenotype analysis of patients with mutations at the same site R645.

**Patient ID**	**1(Lipska-Zietkiewicz et al., 2017)**	**2-I(Liu et al., 2017)**	**2-II(Liu et al., 2017)**	**3(Zivicnjak et al., 2009)**	**Our patient**
Gender	M	F	F	M	M
Country of origin	China	Germany	Germany	France	China
Nucleotide change	c.1933C > T; c.2450G > A	c.1934G > A; c.2542G > T	c.1934G > A; c.2542G > T	c.1933C > G; c.2425G > A	c.1933C > T; c.2141+5G > A
Protein change	p.R645C; p.R817H	p.R645H; p.E848X	p.R645H; p.E848X	p.R645G; p.G809R	p.R645C
Diagnosis	Mild SIOD	Mild SIOD	Mild SIOD	Mild SIOD	Mild SIOD
Diagnosis year	6	11	16	10.8	5
Diagnostic basis	Phenotype	Phenotype	Phenotype	Phenotype	WES
Age at 1st manifestation (years)	5.7	4	6	?	4
1st manifestation phenotype	Proteinuria	Steroid-resistant nephrotic syndrome	Steroid-resistant nephrotic syndrome	?	Hyperthyreosis
Renal function	IgM nephropathy	Renal failure	Terminal renal failure	Renal failure	Asymptomatic proteinuria
Histopathology	Glomerular segmental mesangial matrix hyperplasia	Absence of foot processes and minimal changes in 18 glomeruli	Absence of foot processes and minimal changes in 18 glomeruli	FSGS	Normal
Neurological signs and symptoms	Normal	Normal	Psychosocial problems and reactive depression	Ischemic stroke, demyelinating peripheral neuropathy, epilepsy	Normal
Infections, immunodeficiency	Lymphocytoponia, congenital immune deficiency, decreased blood IgG values	Cyclic lymphopenia, CMV, varicella zoster, helicobacter, and enterococci infections, enterobacteriacea sepsis complicated	High EBV viral load, ocular varicellazoster and herpes encephalitis, sepsis	Catheter-related sepsis SCID	No recurrent infections, decreased ratio of lymphocytes, T cell immunodeficiency
Other extra-renal signs	Puffy eyelids and edema of the lower extremities	Cardiac arrest, papillomas hands	Severe, therapy-resistant papilloma on face, feet and hands	Multiple pigmented nevi, IUGR, preterm delivery	Papilloma on abdomen and bilateral ankle
Thyroid function	Normal	?	?	Normal	Hyperthyreosis
Hypoevolutism	95 cm at 6-years-old (<3rd percentile)	Severe height impairment	Severe height impairment	Height impairment	Short stature
Scoliosis	Scoliosis	Progressive hip dysplasia	Progressive hip dysplasia	Femoral head necrosis	Scoliosis

*FSGS, focal glomerular sclerosis; CMV, cytomegalovirus; EBV, Epstein–Barr virus; SCID, severe combined immune deficiency; IUGR, intrauterine growth retardation.*

### PPI Network, BP Enrichment Analysis, and Renal Expression Profile of *SMARCAL1*-Interacted Genes

**FIGURE 4:**
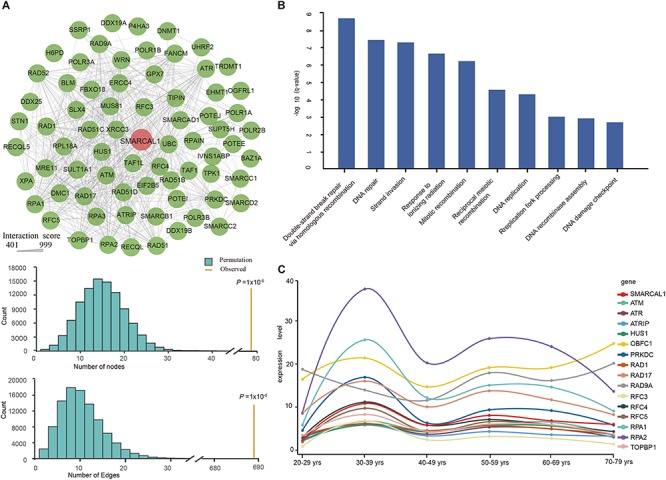
Protein–protein interaction (PPI) Network, Gene Ontology enrichment analysis, and renal expression profile of *SMARCAL1*-interacted genes. **(A)** Interconnected PPI network of the 73 genes with interaction score more than 400 with *SMARCAL1*. A permutation test for genes and connections was conducted with 100,000 iterations. Nodes denote genes, and edges denote interactions between two genes. The thickness of an edge denotes the interaction scores between gene pairs. **(B)** The BP enrichment analysis of the 73 genes in the PPI network. The x-axis shows the GO terms, and the y-axis represents the value of –log10 (*q*-value), indicating the enrichment scores of the GO terms. All *P*-values are corrected for multiple testing using FDR. **(C)** Renal expression profile of 15 genes with the strongest genetic interaction effect with *SMARCAL1*. Different color represents special gene, while red indicates *SMARCAL1.*

Functional interactions between proteins are vital during cellular biological processing and their systematic characterization provides a background of the molecular systems biology. To investigate the PPI network of *SMARCAL1*, we applied relevant data from the STRING v10 database (Szklarczyk et al., 2015). As a result, a total of 73 genes were included in the interconnected PPI network, with an cut-off interaction score greater than 400 (Figure 4A). The PPI network with varying degrees of interactions showed an excess number of interacting proteins among those encoded by the 73 genes (*P* = 1 × 10^–5^) and an excess of pairwise connections (*P* = 1 × 10^–5^) compared with 100,000 randomly simulated datasets (Figure 4A). In this network, two genes *RPA2* and *RAP1* which encode subunits of the heterotrimeric replication protein A (RPA) complex showed the strongest interaction with *SMARCAL1*, with an interaction score of 997 and 974 respectively. In addition, genes encoding ATR–ATRIP protein kinase complex which is crucial for the cellular response to replication stress and DNA damage were also found to have tightly connection to *SMARCAL1.*

To further understand the biological function of these 73 genes, we performed an enrichment analysis of biological processes by DAVID Bioinformatics Resources. The enrichment results showed multiple significant terms with FDR values < 0.05, such as “double-strand break repair via homologous recombination (GO:0000724),” “DNA repair (GO:0006281),” and “replication fork processing (GO:0031297)” (Figure 4B).

Defining the normal expression of *SMARCAL1* gene is an essential first step in determining the cells that contribute to the pathogenesis of FSGS in SIOD patients. To detect the expression profile of *SMARCAL1*-interacting genes in the kidney, we extracted relevant transcriptional data from the GTEx database. The top 15 *SMARCAL1*-interacting genes except *RFC5* showed a similar renal expression profile to *SMARCAL1* (Figure 4C).

## Discussion

Schimke immuno-osseous dysplasia is a rare autosomal recessive disorder caused by compound mutations of *SMARCAL* (Boerkoel et al., 2002) characterized by dysmorphism (Saraiva et al., 1999; Boerkoel et al., 2000), spondyloepiphysial dysplasia (Spranger et al., 1991; Lucke et al., 2006), T cell immunodeficiency (Boerkoel et al., 2000; Lucke et al., 2006), and nephrotic syndrome (Boerkoel et al., 2000; Lucke et al., 2006). SIOD shows phenotypic heterogeneity from mild to severe phenotypes (Simon et al., 2014) and the severity is proportionate to the degree of *SMARCAL1* inactivation (Elizondo et al., 2009). Infantile-onset, early lethal severe patients would carry at least one *SMARCAL1* loss-of-function mutation (deletion, nonsense, or frameshift), whereas patients with a less severe phenotype and survival into the second 10 years would likely carry compound missense mutations (Lou et al., 2002). However, Elizondo et al. reported missense mutations that alter subcellular location, enzymatic activity, protein levels or chromatin binding could result in severe phenotypes (Elizondo et al., 2009). From all of previously reported patients (summarized in Supplementary Table S1), we found that severe SIOD could arise from missense mutations, while frameshift and truncation mutations could lead to a mild phenotype, further suggesting that the genotype–phenotype correlations remain largely unpredictable (Elizondo et al., 2009).

Compared to patients initially diagnosed as SIOD mainly based on a characteristic phenotype, those with SIOD probands diagnosed unexpectedly by genetic screening (SRNS-gene panel) showed a milder extrarenal phenotype during follow-up and a better 10-year patient survival (Lipska-Zietkiewicz et al., 2017). It is known that WES can effectively identify the pathogenic mutations for those whose clinical symptoms are too mild to diagnose the disease, especially rare diseases (Wu et al., 2012; Sarin et al., 2015; Krabbenborg et al., 2016). Notably, the extremely mild and non-characteristic phenotype of our patient did not allow for a definite diagnosis, and thus our patient was the first diagnosed based on a genetic test applying WES before the disease was confirmed. The only previously reported SIOD patient employing WES as genetic testing was conducted after an initial phenotype-based diagnosis accompanied with severe neurodevelopmental delay, facial dysmorphism, and spondyloepiphyseal bone dysplasia (Simon et al., 2014). Our study proves once again that WES is a high-performance tool in the early diagnosis of the extremely rare diseases.

Interestingly, four additional SIOD patients carried missense mutations at the same residue Arg645 (e.g., Arg645Cys, Arg645His, Arg645Gly) (Zivicnjak et al., 2009; Lipska-Zietkiewicz et al., 2017; Liu et al., 2017) accompanied by another different mutation, and two of these were male siblings from a German family (Zivicnjak et al., 2009) (Table 2). All four patients were diagnosed as mild type according to the combined consideration of phenotypes. When reviewing their medical history, an obvious organic lesion of the kidney was observed in the four patients before diagnosis. In contrast to those, our affected patient exhibited asymptomatic proteinuria without recurrent infections or neurological signs and symptoms. Furthermore, the phenotype of the five patients differ from each another, including the two siblings, suggesting sensitive phenotypic pleiotropy. The same *SMARCAL1* mutations have been discovered in both mild and severe cases of the disease, and evidence of environmental factors shaping SIOD phenotypes in fruit flies, mice, and humans (Lucke et al., 2005; Baradaran-Heravi et al., 2012) has been found. Thus, the effect of a *SMARCAL1* mutation cannot only be determined by the *SMARCAL1* genotype but also by the stochastic, environmental, and genetic background variations (Lucke et al., 2005; Carroll et al., 2013).

The patient described in this study harbored a novel splicing mutation (c.2141+5G > A) confirmed to be an alternate donor splice site mutation, resulting in the loss of C-terminal functional region of SMARCAL1 protein. Moreover, the novel mutation was classified as a “pathogenic variant” according to the guide of ACMG. Similarly, a reported SIOD patient with mild developmental delay and mild ID carried a homozygous mutation c.2244+5 G > A (Lipska-Zietkiewicz et al., 2017), within the donor site of intron 14. Both the two splicing sites are very close to the initial amino acid encoding the helicase C-terminal region, in which most disease-related mutations are accumulated. Phosphorylation of subdomain in the C-terminus can change SMARCAL1 activity, resulting in either overexpression or silencing of *SMARCAL1* that then causes the accumulation of replication-associated DNA damage (Carroll et al., 2014).

*SMARCAL1* showed the strongest interaction with genes encoding RAP complex and ATR–ATRIP protein kinase complex in the PPI network (Figure 4A). SMARCAL1 is an ATP-driven annealing helicase that catalyzes the formation of double-stranded DNA from complementary single-strand DNA strands by binding to *RPA* (Yusufzai and Kadonaga, 2008; Yusufzai et al., 2009). RPA complex, which is involved in DNA replication, repair, recombination, telomere maintenance, and coordinating the cellular response to DNA damage, plays an important role in DNA metabolism (Liaw et al., 2011; Daniels et al., 2012). The recruitment of ATR–ATRIP complex at sites of DNA damage is based on RPA-coated ssDNA, which facilitates complex recognition of substrates for phosphorylation and the initiation of checkpoint signaling (Zou and Elledge, 2003). In the developing fetal kidney, SMARCAL1 is expressed in the ureteric epithelium, stroma, metanephric mesenchyme, and in all stages of the developing nephron, including the maturing glomerulus, suggesting an important, functional role for the SMARCAL1 helicase during kidney development (Dekel et al., 2008). The absence or disruption of SMARCAL1 protein function in SIOD patients might cause abnormalities in kidney development that manifested during the perinatal or adolescent period. In the postnatal adult kidney, SMARCAL1 is expressed in the glomerulus, tubules of the nephron, and collecting ducts, demonstrating a role for SMARCAL1 protein in the maintenance and integrity of podocytes and endothelial cells (Sarin et al., 2015). Most of the genes showed strongest interaction with *SMARCAL1* showing a similar renal mRNA expression profile to *SMARCAL1* that indicates a similar role these genes play in renal function maintenance.

## Conclusion

We reported a 5-year-old girl with an extremely mild phenotype based on genetic testing by applying WES. We identified a new allelic mutation (c.2141+5G > A) of *SMARCAL1*, which is confirmed to create a novel splice donor site. Our results provide new insights into the phenotypic spectrum of *SMARCAL1*. The biological function and renal expression analyses of *SMARCAL1*-interacting genes provide additional genetic evidence supporting the potential role of *SMARCAL*1 in SIOD.

## Ethics Statement

This study was conducted in accordance with the guidelines of the Declaration of Helsinki. It was also approved by the Ethics Committee of the Second Affiliated Hospital and Yuying Children’s Hospital of Wenzhou Medical University. Written informed consent of all participants was collected from their parents or guardians at the time of recruitment for participation in the study and publication of the study.

## Author Contributions

JJ was involved in the study design, data analysis, and manuscript drafting. ZL and XC performed exome sequencing. SJ performed Sanger sequencing. KW participated in the data analysis and performed clinical evaluation of the patients. ZW and WL were involved in the study design and critical evaluation of the manuscript. All authors read and approved the final manuscript.

## Conflict of Interest Statement

The authors declare that the research was conducted in the absence of any commercial or financial relationships that could be construed as a potential conflict of interest.

## Footnotes

1 https://david.ncifcrf.gov/summary.jsp
