# Supplementary material for: Whole Exome Sequencing Identified a Novel Biallelic SMARCAL1 Mutation in the Extremely Rare Disease SIOD
Source: Front Genet. 2019 Jun 18;10:565. doi: 10.3389/fgene.2019.00565 (PMC6591458; doi:10.3389/fgene.2019.00565)
Supplement: Supplementary file 1 [file Data_Sheet_1.PDF]

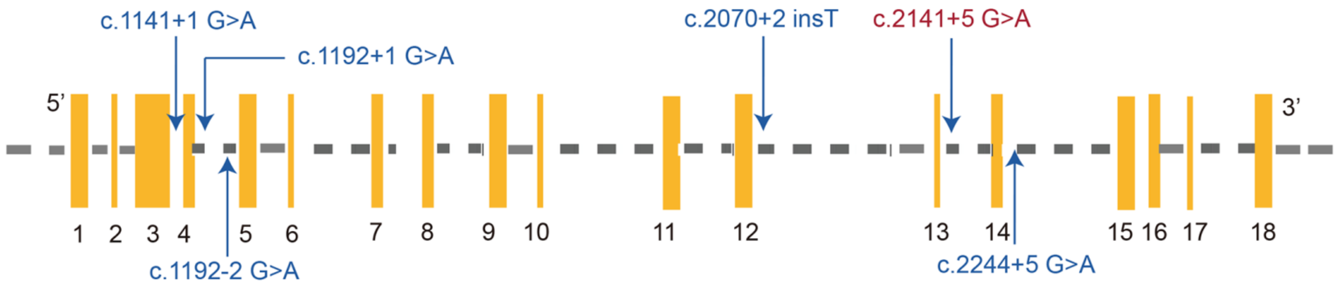

Supplementary Figure S1 Nucleotide structure diagram of all the splicing mutations detected in SIOD patients. Yellow boxes represent the coding exons; black characters indicate the mutation identified in this study.

TableS1 Phenotypic profile and mutation summary of reported SIOD patients

Mutation	Ethnicity	Sex	Age (y)	Spondylo-epiphyseal dysplasia	Dysmorphic facial features	Macules	Renal disease	Lympho-cytopenia	blood pancytopenia	recurrent infection	CNS (migraine headaches, cerebral ischemia）	Positive sign	Reference (PMID)
L397fsX40; E848X	Irish/Scottish	M	8	+	+	+	+	+	+	+	+	8	11799392
L397fsX40; ?	Caucasian	F	5.7	+	+	+	+	+	+	+	–	7	11799392
Del exons 1–4	English/ Scottish/Irish/ Hungarian	M	3.7	+	+	+	+	+	+	–	+	7	11799392
Del exons 1–4	from Indian	F	9	+	+	+	+	+	+	+	–	7	11799392
E848X; R764Q	German	M	10.5	+	+	+	+	+	+	+	–	7	11799392
c.1141+1 G>A; 645fsX17	Iraqi	F	10	+	+	+	+	+	–	+	+	7	11799392
?; R820H	Irish/French/	M	10.6	+	+	+	+	+	+	+	–	7	11799392
S774X; S579L	Portuguese	M	8	+	+	+	+	+	+	+	–	7	11799392
R820H	Welsh	M	14	+	+	+	+	+	+	–	+	7	11799392
R17X; Q34X	Welsh	F	9.6	+	+	+	+	+	–	+	+	7	11799392
M1?; R561H	China	M	8	+	+	+	+	+	+	–	–	6	20179009

E848X	Czechoslovakian/ German/ Hungarian/ Ukranian	F	6.4	+	+	+	+	+	–	–	+	6	11799392
c.2070+2insT; I821S	France	M	9.8	?	–	+	+	+	+	+	+	6	28796785
V568fsX1	France	M	4	+	+	–	+	+	–	+	+	6	11799392
E398X	from Indian	F	14	+	+	+	+	+	–	–	+	6	11799392
Q568fsX3; R644W	German	M	8	+	+	+	+	+	–	+	–	6	11799392
Q568fsX3; R644W	German	F	8	+	+	+	+	+	–	+	–	6	11799392
c.1192+1 G>A	German	M	10.5	+	+	+	+	+	–	+	–	6	11799392
A468P; T705I	German	M	15	–	+	+	+	+	–	+	+	6	11799392
R820H; E848X	Span	F	5.9	+	+	+	+	+	–	–	+	6	11799392
641fsX50; S774X	Span	M	6.8	+	+	+	+	+	–	+	–	6	11799392
M566F	Turkey	M	11.7	+	+	+	+	+	–	+	–	6	11799392
K647Q	Turkey	F	5.8	+	+	+	+	+	–	–	+	6	11799392
H379P；F279S	?	M	?	+	+	+	+	+	–	+	–	5	15884045
K647T	Algeria	F	19.8	+	+	+	+	+	–	–	–	5	11799392
G461D	Czech	M	11.5	?	–	+	+	+	–	+	+	5	28796785

Del exons 1–4	English/ Scottish/Irish/ Hungarian	F	2.8	+	+	+	+	+	–	–	–	5	11799392
E848X	Finnish	M	8.5	+	+	–	+	+	–	–	+	5	11799392
Y342X; I755S	Italy	M	4.6	?	–	+	+	+	–	+	+	5	28796785
Q770X	Jordan	M	6	?	–	+	+	+	–	+	+	5	28796785
E848X	Poland	F	10.1	?	–	+	+	+	–	+	+	5	28796785
I548N; R645C	Scottish/French	M	25.9	+	+	+	+	+	–	–	–	5	11799392
c.1192-2 G>A	Ashkenazi	M	6	+	–	+	+	+	–	–	–	4	18356746
P480L; E848X	Czech	F	5.7	?	–	+	+	+	–	–	+	4	28796785
R645G; G809R	France	M	13.1	?	–	+	+	–	–	+	+	4	28796785
c.2070+2insT; I821S	France	M	10	?	–	+	+	–	–	+	+	4	28796785
L718SfsX13; E848X	German	M	3.4	?	–	+	+	+	–	+	–	4	28796785
E848X; R645H	German	F	16	+	–	–	+	+	–	+	–	4	19127206
836T>C;2542G>T	German	M	16	+	–	+	+	–	–	–	+	4	15880370
exon 10 – ins1849C;	Israel	F	4	+	+	–	?	+	–	+	–	4	24197801
S579X	Lebanon	M	6.6	?	–	–	+	+	–	+	+	4	28796785
F279S; E848X	Poland	F	9.1	?	–	–	+	+	–	+	+	4	28796785
W620X; E848X	Poland	M	11.2	?	–	+	+	+	–	+	–	4	28796785
R644Q; E848X	Poland	M	8.3	?	–	+	+	+	–	+	–	4	28796785

E848X	Poland	M	6.1	?	–	+	+	+	–	+	–	4	28796785
L397fxX40; S774X	Portuguese	M	9.3	+	+	–	+	+	–	–	–	4	11799392
L539V; L539_IIe548del	Spain	M	4	+	–	–	+	+	–	+	–	4	27282802
R586W	Turkey	M	18.1	?	–	+	+	+	–	+	–	4	28796785
K647T	Algeria	F	6.6	?	–	–	+	+	+	–	–	3	28796785
L397fsX40; S859P	Caucasian	M	6	+	?	?	+	+	–	–	–	3	23630135
R817H; R645C	China	M	6	+	–	–	+	+	–	–	–	3	29282041
R17X; F279S	German	F	15.9	?	–	–	+	+	–	–	+	3	28796785
K100X; T705I	German	F	16	?	–	+	+	+	–	–	–	3	28796785
F279S; E848X	German	M	31.2	?	–	–	+	+	–	–	+	3	28796785
E848X; del (2) (q34–q36)	German	F	6.4	?	–	–	?	+	+	–	+	3	28796785
E848X; R645H	German	F	11	?	–	–	+	+	–	+	–	3	19127206
836T>C; 2542G>T	German	M	?	+	–	+	+	–	–	–	–	3	15880370
exon 10 – ins1849C; exon 12 – del2161C stop codons	Israel	M	1	+	+	–	+	?	?	?	–	3	24197801
R586W	Italy	M	36.3	+	–	–	+	+	–	–	–	3	11799392
R586W	Italy	F	23.9	+	–	–	+	+	–	–	–	3	11799392

P480L; I755S	Italy	F	9	?	–	–	+	+	–	+	–	3	28796785
S579X	Lebanon	F	6.8	?	–	–	+	+	–	+	–	3	28796785
S579X	Lebanon	M	6.3	?	–	+	+	+	–	–	–	3	28796785
G580V; P808R	Macedonia	M	7.3	?	–	–	+	–	–	+	+	3	28796785
K647T	Morocco	F	6.7	?	–	–	+	+	–	–	+	3	28796785
R764W	Saudi Arabia	M	10.7	?	–	+	+	?	–	–	+	3	28796785
R820H	Turkey	F	8	?	–	+	+	–	–	+	–	3	28796785
R561C	Turkey	M	10	+	?	?	+	+	–	–	–	3	16237566
R17X	German	F	7.45	?	–	+	?	–	–	–	+	2	28796785
R764W	Saudi Arabia	F	7.2	?	–	+	+	?	–	–	–	2	28796785
R820H	Turkey	M	9.2	?	–	–	+	?	–	+	–	2	28796785
F279S; E848X	German	M	34.1	?	–	–	+	?	–	–	–	1	28796785
V736GfsX75	Saudi Arabia (Yamani)	M	10.4	?	–	–	+	–	–	–	–	1	28796785
R561C	Turkey	F	18.6	?	–	–	+	–	–	–	–	1	28796785
R820H; S625X	Norwegian	M	?	?	?	?	?	?	?	?	?	0	11799392
c.2244+5G>A	United Kingdom	M	5.5	?	–	–	?	?	–	–	?	0	28796785

Table S2 ACMG assessment of the p.Arg645Cys and c.2141+5G>A variants in *SMARCAL1*

Mutation	Functional prediction		Frequency		ACMG Classification	Evidence	
	SIFT	Polyphen-2	1000G	ExAC		Criteria	Implication
c.C1933T	Damaging (0)	Probably Damaging (1)	_	_	Pathogenic	PS1	Same amino acid change as a previously established pathogenic variant regardless of nucleotide change
						PS3	Well-established in vitro or in vivo functional studies supportive of a damaging effect on the gene or gene product
c.2141+5G>A	_	_	_	_	Pathogenic	PVS1	Null variant (nonsense, frameshift, canonical ±1 or 2 splice sites, initiation codon, single or multiexon deletion) in a gene where LOF is a known mechanism of disease
						PM2	Absent from controls (or at extremely low frequency if recessive) in Exome Sequencing Project,1000 Genomes Project, or Exome Aggregation Consortium
						PM3	For recessive disorders, detected in trans with a pathogenic variant

'_' represents the information was unavailable in the database.

Table S3 Splice-alternation of c.2141+5 G >A predicted by splicing prediction tools

**Splicing Prediction Tools**	**Prediction Algorithm**	**Prediction Score**	**Prediction Result**	**Evaluation Criterion**
dbscSNV (https://sites.google.com/site/jpopgen/dbNSFP.)	Adaboost	0.999	splice-altering	define a variant to be splice-altering if either its ada score or rf score was larger than 0.6
	Random Forests	0.964		
Human Splicing Finder (http://www.umd.be/HSF3/)	HSF Matrics	–	alternation of the WT donor site, most probably affecting splicing	_
	MaxEnt	_		
MaxEntScan (http://genes.mit.edu/burgelab/maxent/Xmaxentscan_scoreseq.html)	Maximum entropy modeling (wide type)	8.31	the mutation might disrupt 5'-splice site function and result in the skipping of exon 13 from the mature mRNA	the mutation might disrupt splice site function and result in the skipping of exon if the score after mutation is reduced by more than 15% before the mutation
	Maximum entropy modeling (mutation)	2.07		
SPANR (http://tools.genes.toronto.edu/)	"deep learning" computer algorithms	-3.84 (dPSI: the percent spliced in)	have a effect on splicing	difference in the percentage of transcripts with the exon spliced in [dPSI] score of -3.84

'_' represents the information was unavailable.
